# The diagnostic capability of electrocardiography on the cardiogenic shock in the patients with acute myocarditis

**DOI:** 10.1186/s12872-020-01796-4

**Published:** 2020-12-01

**Authors:** Dan Yang, Qing Dai, Han Wu, Jianzhou Chen, Jingmei Zhang, Zhonghai Wei

**Affiliations:** 1 Department of Cardiology, Drum Tower Hospital, Medical School of Nanjing University, Nanjing, 210008 China; 2 Department of Cardiology, Yizheng Hospital, Nanjing Drum Tower Hospital Group, Yizheng, 211900 China

**Keywords:** Fulminant myocarditis, Cardiogenic shock, Electrocardiography, Diagnosis

## Abstract

**Background:**

The study was performed to assess the diagnostic capability of ECG on the cardiogenic shock (CS) in acute myocarditis. A new score was derived from the combination of the ECG parameters and the diagnostic value was also evaluated.

**Methods:**

Total 103 consecutive patients with acute myocarditis admitted in Nanjing Drum Hospital were enrolled in the current study. The cohort was divided into fulminant myocarditis group (FM, n = 20) and non fulminant myocarditis group (NFM, n = 83). The demographic features, results of electrocardiography (ECG) and ultracardiography were compared. Logistic regression analysis was conducted to identify the relevant factors in ECG parameters. We created a new variable called “ECG score” by certain combination of ECG parameters. The diagnostic capability of ECG score for CS was compared with the existing diagnostic indices using regression model and receiver-operating characteristics (ROC) analysis.

**Results:**

There were several changes on ECG significantly different between the two groups. Multivariate regression analysis demonstrated PR + QRS interval (*P* = 0.008), ventricular arrhythmia (*P* = 0.001) and pathological Q wave (*P* = 0.003) were the independent relevant factors of CS. The derived variable “ECG score” was identified as a significant relevant factor of CS by multivariate regression model. ROC analysis showed PR + QRS interval, ventricular arrhythmia and pathological Q wave all had equivalent diagnostic capability to left ventricular ejection fraction (LVEF) and shock index (SI). ECG score was equivalent to LVEF but superior to SI in diagnosing CS

**Conclusions:**

ECG was valuable in diagnosing CS due to acute myocarditis. The ECG score was superior to the traditional diagnostic indices and could be used for an rapid recognition of CS.

## Background

Myocarditis is a inflammatory disease involving injury of the cardiac myocytes, the incidence of which is estimated approximate 0.02–0.1% in the general population [1, 2]. Fulminant myocarditis (FM), the most severe type, is characterized with cardiogenic shock (CS) and haemodynamical disorder. It usually undergoes a fatal course and some of the cases probably come to a frustrating end. On the contrary, nonfulminant myocarditis (NFM) often produces symptoms of heart failure with stable haemodynamical status [3]. Of note, NFM could sometimes evolve into FM very quickly after first medical contact (FMC). It has been reported that FM patients have more likelihood of death or heart transplantation [4, 5]. Aggressive treatment including inotropic agents, mechanical circulatory support would help the patients get through the CS stage and improve the prognosis [6]. Thus, quick recognition of the upcoming or existing CS is essential.

As far as we know, CS has an arbitrary definition of systolic blood pressure below 90 mmHg with inadequate peripheral perfusion. The diagnostic accuracy is influenced by the basic level of the blood pressure. Shock index (SI) is a more widely used for prediction or diagnosis of shock. Different cutoff values have been suggested, which reveals that the diagnostic capability of SI probably varies under different clinical conditions [7–10]. Therefore, more diagnostic measures are needed for diagnosing the CS in the presence of myocarditis.

It has been observed that the different components of electrocardiography (ECG) usually alter in myocarditis patients, such as prolonged PR interval, wide QRS complex, nonspecific ST-T changes, emergence of pathological Q wave [11]. Although many changes are not pathognomonic, a few features are more likely to occur in the FM than NFM [12–15]. Based on the above findings, we postulate that combination of the various alterations on the ECG might create an useful index for rapid diagnosis of the CS in acute myocarditis patients.

## Methods

### Study population

All the patients who were suspected of acute myocarditis were admitted in Nanjing Drum Hospital, Medical School of Nanjing University from November 2010 to September 2019. The inclusion criteria were as follows: (1) The included paitents had no age limitation; (2) The symptom onset was less than 1 week before admission; (3) The diagnosis of acute myocarditis was confirmed after clinical, laboratory tests and imaging evaluation; (4) The whole data were available. The exclusions criteria were as follows: (1) The symptom onset was above 1 week before addmission; (2) The myocardial injury was due to other heart diseases, such as worsening of heart failure, acute coronary syndrome (ACS), arrhythmia, cardiomyopathy; (3) The myocardial injury was caused by other systemic diseases, such as tumor, immune disorder, hematological disease, stroke; (4) The patients suffered from acute pericarditis without myocardial injury; (5) The clinical data were not accessible.

**Fig. 1:**
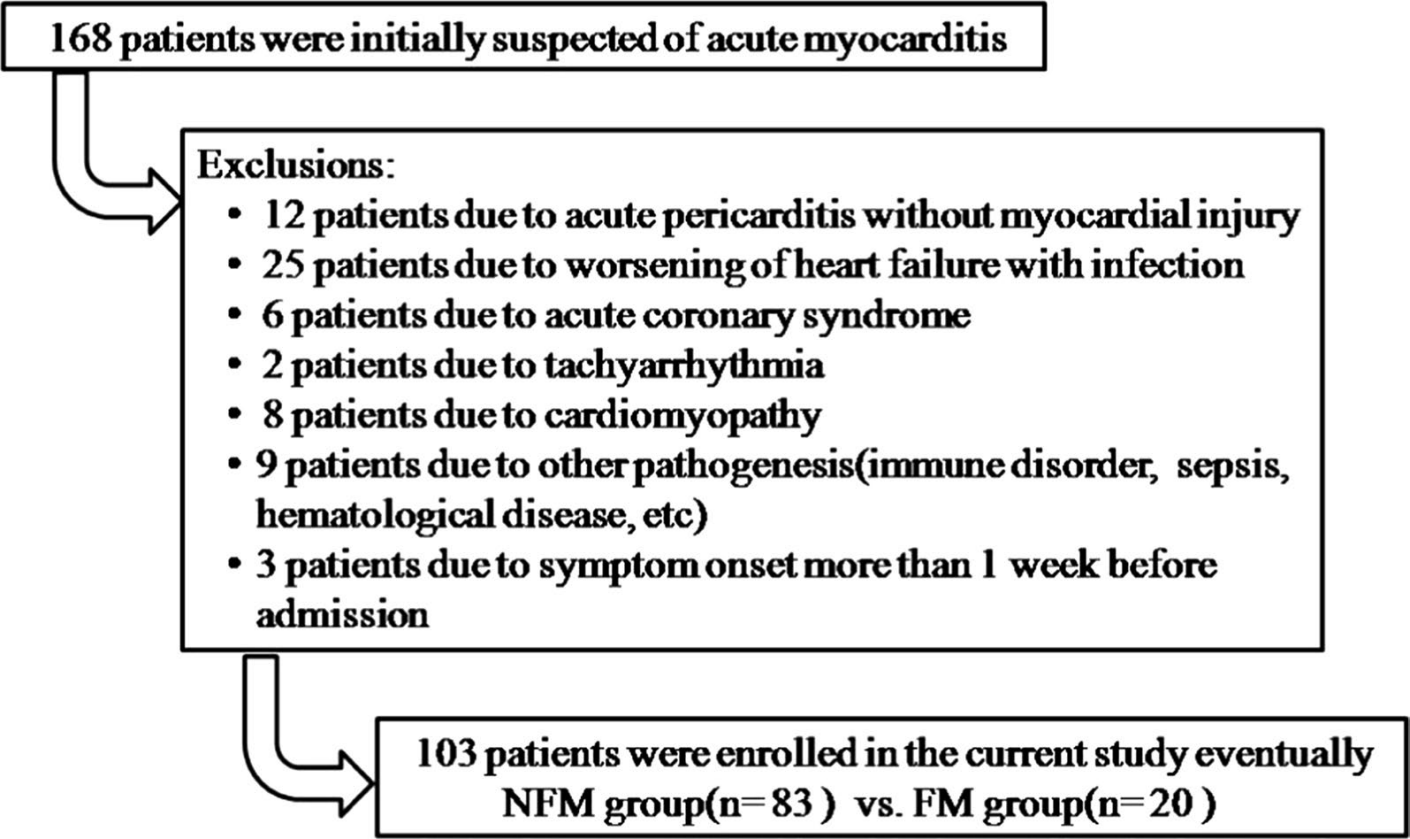
Flow chart describing the enrollment of the patient cohort. *NFM* nonfulminant myocarditis, *FM* fulminant myocarditis

Total 168 patients suspicious of acute myocarditis were put into initial analysis. Acute myocarditis was diagnosed based on the following clinical presentations and auxiliary tests [16]: (1) the history of flu-like prodromes including respiratory or gastrointestinal symptoms; (2) acute onset of symptoms such as chest discomfort, palpitation, shortness of breath, fatigue, etc.; (3) elevation of myocardial injury biomarkers; (4) various changes on ECG including non-specific ST-T alteration, ACS pattern, pericarditis pattern, atrioventricular block (AVB), etc.; (5) structure or function abnormality on ultracardiography (UCG); (6) exclusion of coronary heart disease (CHD) or present CHD not responsible for the above manifestations. Besides, cardiac magnetic resonance (CMR) was performed for some patients after consideration of the individual conditions. CMR could provide the pathognomonic features for confirming the diagnosis and evaluation of the myocardial injury. FM could be diagnosed when the cardiac function was severely impaired which led to CS with or without fatal ventricular arrhythmia [17]. CS was defined as systolic blood pressure less than 90 mmHg for at least 30 min, which presented clinical signs of pulmonary congestion and peripheral hypoperfusion or needed inotropic agents to maintain the blood pressure level. After series of evalutions, 12 patients were exluded due to pericarditis without myocardial injury. 41 patients were excluded due to other heart diseases (ACS, tachyarrhythmia, heart failure, etc.). 9 patients were excluded due to other systemic disease (immune disorder, stroke, hematological diseases, etc.). 3 patients were excluded due to the symptom onset more than 1 week before admission. Finally, 103 patients were eligible for the study cohort (Fig. 1).

The study cohort had a median age of 30 years (IQR: 22–46 years) and contained 40 female patients (38.8%) and 63 male patients (61.2%). There were 20 patients (19.4%) with diagnosis of FM and the rest were NFM. The study cohort was divided into FM group and NFM group for further analysis. Data was obtained from the databases in our institution and the ethics has been approved by the Medical Ethics Committee of Nanjing Drum Tower Hospital, Medical School of Nanjing University (2019-190-01).

### Patient management

All the patients were admitted into cardiac care unit (CCU) for monitoring the potential adverse cardiac events. Bedside UCG was accomplished within 24 h after hospitalization. If the patients were suspicious of ACS according to the risk factors, symptom, alterations on ECG, etc., angiography was performed after admission. CMR was taken if necessary for confirming the diagnosis or assessing the extent of myocardial injury. If the patients had symptoms of cardiac dysfunction but with haemodynamical stability, the diuretics, β adrenergic blockade, angiotensin-converting enzyme inhibitor (ACEI)/angiotensin receptor blockade (ARB) and aldosterone antagonist were considered for individual therapy [18]. As to the patients with fulminant type, measurements for reversing the haemodynamical derangement were carried out as soon as possible. Intra-aortic balloon pump (IABP) was the first option of mechanical circulatory support. The inotropic agents were considered in combination with IABP. Noninvasive respiratory ventilation was utilized for alleviating the symptoms of dyspnea due to pulmonary edema. If the haemodynamical status could not be stabilized after the support of IABP and noninvasive ventilation, extracorporeal membrane oxygenation (ECMO) and invasive ventilation were performed. Swan-Ganz catheter was deployed for haemodynamical monitor according to the individual conditions. The temporary pacemaker was inserted if there was second to third degree AVB. Prolonged PR interval combined with wide QRS complex on the ECG might suggest a high likelihood of upcoming complete heart block, prophylactic temporary pacemaker was therefore used. Ventricular tachycardia (VT) was life-threatening in fulminant myocarditis. Defibrillation and/or overdriving by temporary pacemaker were helpful for termination of sustained VT and aminodarone was administrated for control of the malignant ventricular tachycardia.

### Statistical analysis

Continuous variables were shown as mean ± standard deviation (mean ± SD) if they were normally distributed, whereas they were presented as median and interquartile range (IQR) if they were skewed distributed. Categorical variables were shown as frequencies and percentages. The comparison of the continuous variables between the two groups was performed using T-test or Wilcoxon rank-sum test. The comparison of the categorical variables between the two groups was performed using χ^2^ test or Fisher exact test. The potential predictors of CS, selected from demographic features, ECG parameters and UCG parameters, were analyzed using univariate logistic regression initially. Considered the interest of ECG changes in the current study, we picked up the ECG parameters with *P* < 0.10 for multivariate logistic regression analysis in order to identify the significant relevant factors. After the multivariate analysis, the significant ECG parameters were integrated to derive a new variable (we called ECG score). This derived ECG score together with other covariates were set in the multivariate logistic regression model for evaluation of the odd ratio. In the multivariate regression model, the variables were selected using backwards method. The regression models were calibrated with Hosmer–Lemeshow χ^2^ test for the goodness of fit. The correlation analysis was carried out using Pearson coefficient (r) or Spearman coefficient (ρ) for the colinearity tests of the covariates. The diagnostic capability of the significant variables was tested with receiver-operating characteristics (ROC) analysis. The area under curve (AUC) was calculated to compare the diagnostic capability of the ECG score with the traditional diagnostic indices. Specificity, sensitivity, accuracy, positive predictive value (PPV) and negative predictive value (NPV) were analyzed for identification of a reasonable cutoff value. The statistical analysis was performed by Stata version 12.0 (StataCop., College Station, Texus, USA). All the tests were 2 sided. Values of *P* < 0.05 were considered statistically significant.

## Results

### The demographic characteristics of the study cohort

The median age of the patients in the FM group was numerically higher than that of the patients in NFM group with a trend towards statistical significance. In the FM group, female patients accounted for a much higher proportion. Meanwhile, 17 patients (85%) had presented CS when admitted in the emergency department (ED), while 3 patients (15%) developed CS on the second day after hospitalization. FM patients had also higher level of myocardial injury biomarkers, inflammatory biomarkers, brain natriuretic peptide (BNP) and hepatic transaminase (Table 1).

**Table 1:** The baseline characteristics of patient cohort

	FM group (n = 20)	NFM group (n = 83)	*P* value
Age (year)	36 (29–48)	28 (22–44)	0.06
Female	12 (60%)	28 (33.7%)	0.03
Hypertension	1 (5%)	8 (9.6%)	1.00
Diabetes	0	3 (3.6%)	1.00
Coronary heart disease	0	1 (1.2%)	1.00
Smoker	4 (20%)	9 (10.8%)	0.27
Drinker	1 (5%)	6 (7.2%)	1.00
BMI (kg/m^2^)	24.4 ± 2.87	23.0 ± 3.19	0.15
Onset symptom			
Chest discomfort	15 (85%)	64 (77.1%)	0.55
Fatigue	6 (30%)	12 (14.5%)	0.11
Palpitation	2 (10%)	14 (16.9%)	0.73
Cough	5 (25%)	11 (13.3%)	0.30
Diarrhea	2 (10%)	11 (13.3%)	1.00
Onset to FMC (day)	3 (3–4)	3 (2–6)	0.62
Cardiogenic shock			< 0.0001
Presented when admission	17 (85%)	0	
Developed after admission	3 (15%)	0	
IABP use	17 (85%)	0	< 0.0001
ECMO use	6 (30%)	0	< 0.0001
Temporary pacemaker use	11 (55%)	6 (7.2%)	< 0.0001
Hospitalization stay (day)	15 (10–24)	9 (7–12)	0.003
Fever	12 (60%)	42 (50.6%)	0.31
Temperature (℃)	36.5 (36.1–37.2)	36.5 (36.3–36.9)	0.73
SBP (mmHg)	90.5 ± 9.70	117.6 ± 15.8	< 0.0001
DBP (mmHg)	61.7 ± 12.50	71.2 ± 9.78	0.0004
HR (beats/min)	95.1 ± 23.60	88.7 ± 17.25	0.17
SI	1.0 (0.82–1.81)	0.74 (0.63–0.91)	< 0.0001
RR (per min)	20 (19–25)	20 (19–20)	0.37
WBC (× 109/L)	8.1 (7.1–11.1)	7.3 (5.6–9.3)	0.09
Neutrophil (%)	76.2 ± 10.51	68.8 ± 11.88	0.01
Lymphocyte (%)	15.4 ± 9.17	21.0 ± 9.07	0.02
BNP (pg/ml)	686 (360–1090)	78 (27–643)	0.0001
Peak CKMB (U/L)	80 (40–115)	32 (14–52)	0.0001
Peak TnT (ug/L)	4.41 (2.59–10)	0.63 (0.23–1.44)	< 0.0001
CRP (mg/L)	64.0 (29.7–112.2)	12 (3.6–63.3)	0.006
ESR (mm/h)	34 (23–55)	27 (9–39)	0.22
ALT (U/L)	184 (56–1109)	31 (22–60)	< 0.0001
AST (U/L)	302 (69–1197)	47 (28–87)	< 0.0001
TB (umol/L)	12.1 (6.9–16.6)	12.1 (8.8–15.9	0.58
DB (umol/L)	4.4 (2.3–6.8)	3.6 (2.6–4.7)	0.28
BUN (mmol/L)	7.0 (4.8–14.2)	4.2 (3.5–5.3)	< 0.0001
sCr (umol/L)	73 (53–152)	65 (56–76)	0.19
UA (umol/L)	361 (325–532)	340 (308–413)	0.17

The continuous data were presented as mean ± standard deviation or median (interquartile range)

The categorical data were presented as frequency (percentage)

*BMI* body mass index, *IABP* intra-aortic balloon pump, *ECMO* extracorporeal membrane oxygenation, *SBP* systolic blood pressure, *DBP* diastolic blood pressure, *HR* heart rate, *SI* shock index, *RR* respiratory rate, *WBC* white blood cell count, *BNP* brain natriuretic peptide, *CKMB* creatinine kinase MB subtype, *TnT* troponin T, *CRP* C-reactive protein, *ESR* erythrocyte sedimentation rate, *ALT* alanine transaminase, *AST* aspartate transaminase, *TB* total bilirubin, *DB* direct bilirubin, *BUN* blood urea nitrogen, *sCr* serum creatinine, *UA* uric acid

### ECG and UCG findings of the study cohort

The PR intervals and QRS complex duration in the FM group were prolonged significantly than those in NFM group. There were also more patients in the FM group had pathological Q wave and ST-T alterations. Ventricular arrhythmia, including frequent non-sustained VT, sustained VT and accelerated idioventricular rhythm (AIVR) was overwhelming in the FM patients. As to AVB, the incidence of first and third degree AVB was much higher in FM patients (Table 2). Of note, the three patients, who developed CS on the second day after hospitalization, presented nearly normal left ventricular systolic function but abnormal ECG on the first day. One patient had prolonged PR interval, pathological Q wave and non-sustained VT. Another one had prolonged PR interval and wide QRS complex. The last one had prolonged PR interval, wide QRS complex and pathological Q wave.

**Table 2:** ECG and UCG characteristics of patient cohort

	FM group (n = 20)	NFM group (n = 83)	*P* value
ECG parameters			
PR interval (ms)	189 (156–210)	154 (136–174)	0.0006
QRS complex duration (ms)	104 (89–133)	90 (82–100)	0.005
Wide QRS complex	14 (70%)	7 (8.9%)	< 0.0001
QTc interval (ms)	432 (376–464)	401 (387–421)	0.14
QRS-T angle (degree)	51 (35–97)	34 (22–75)	0.08
Pathological Q wave	15 (75%)	9 (11.5%)	< 0.0001
ST segment depression	8 (40%)	8 (9.6%)	0.001
ST segment elevation	12 (60%)	16 (19.3%)	0.003
T wave inverse	16 (80%)	19 (22.9%)	< 0.0001
Ventricular arrhythmia	10 (50%)	5 (6.3%)	< 0.0001
Sinus arrest	1 (5%)	4 (5.1%)	1.00
First degree AVB	10 (50%)	5 (6.3%)	< 0.0001
Second degree AVB	1 (5%)	2 (2.5%)	0.50
High degree AVB	2 (10%)	2 (2.5%)	0.18
Third degree AVB	8 (40%)	6 (7.6%)	0.001
UCG parameters			
IVST (cm)	0.9 (0.8–1.0)	0.8 (0.8–0.9)	0.07
LVPWT (cm)	0.9 (0.8–1.0)	0.8 (0.8–0.9)	0.07
LAD (cm)	3.6 ± 0.51	3.5 ± 0.40	0.50
AoD (cm)	2.6 ± 0.27	2.8 ± 0.29	0.18
LVEDD (cm)	5.0 ± 0.51	5.0 ± 0.44	0.90
LVESD (cm)	4.1 ± 0.67	3.6 ± 0.59	0.001
LVEF (%)	37 (29–46)	57 (50–60)	< 0.0001
PAP (mmHg)	30 (23–33)	25 (21–28)	0.04

The continuous data were presented as mean ± standard deviation or median (interquartile range)

The categorical data were presented as frequency (percentage)

*ECG* electrocardiography, *UCG* ultracardiography, *AVB* atrioventricular block, *IVST* intraventricular septal thickness, *LVPWT* left ventricular posterior wall thickness, *LAD* left atrial diameter, *AoD* aorta diameter, *LVEDD* left ventricular end-distolic diameter, *LVESD* left ventricular end-systolic diameter, *LVEF* left ventricular ejection fraction, *PAP* pulmonary artery pressure

The ventricular wall thickness seemed to be higher in the FM group numerically with a trend towards statistical significance. The left ventricular end-diastolic diameter (LVEDD) was similar while the left ventricular end-systolic diameter (LVESD) was significant lower in the NFM group. The FM patients had a worse left ventricular ejection fraction (LVEF) due to the severe impaired cardiac function (Table 2).

### Establishment of logistic regression model

The univariate logistic regression analysis was carried out for identification of the potential relevant factors for CS. The significant variables were listed in the Table 3. Of note, two groups of variables had colinearity, LVEF and LVESD (ρ = − 0.76, *P* < 0.01), intraventricular septal thickness (IVST) and left ventricular posterior wall thickness (LVPWT) (ρ = 0.95, *P* < 0.01). Thus, LVESD and LVPWT were not used for regression analysis.

**Table 3:** Univariate logistic analysis for CS

Variables	Odd ratio	95% CI	*P* value
Age	1.02	[0.99 1.05]	0.13
Sex (Male:0, Female:1)	2.94	[1.08 8.04]	0.04
BMI	1.15	[0.95 1.40]	0.15
Hypertension	0.49	[0.06 4.19]	0.52
Smoker	2.06	[0.56 7.51]	0.28
Drinker	0.68	[0.08 5.95]	0.72
BMI	1.15	[0.95 1.39]	0.15
SI (per 0.1 increase)	1.06	[1.26 2.04]	< 0.0001
CRP (per 1.0 mg/L increase)	1.01	[0.99 1.01]	0.06
PR interval (per 10 ms increase)	1.29	[1.10 1.51]	0.002
QRS complex duration (per 10 ms increase)	1.59	[1.22 2.06]	0.001
QTc interval (per 10 ms increase)	1.11	[0.98 1.27]	0.11
QRS-T angle (per 10 degree increase)	1.07	[0.97 1.19]	0.19
Pathological Q wave	23.00	[6.74 78.5]	< 0.0001
ST segment elevation	6.28	[2.20 17.9]	0.001
ST segment depression	6.25	[1.97 19.8]	0.002
T wave inverse	13.50	[4.02 45.2]	< 0.0001
Ventricular arrhythmia	14.80	[4.20 52.2]	< 0.0001
Second to Third degree AVB	3.93	[1.60 9.68]	< 0.01
IVST (per 0.1 cm increase)	1.34	[0.98 1.82]	0.06
LVEF (per 5% increase)	0.49	[0.36 0.67]	< 0.0001
PAP	1.09	[0.99 1.19]	0.06

*CS* cardiogenic shock, *CRP* C-reactive protein, *BMI* body mass index, *SI* shock index, *AVB* atrioventricular block, *IVST* intraventricular septal thickness, *LVEF* left ventricular ejection fraction, *PAP* pulmonary artery pressure

**Fig. 2:**
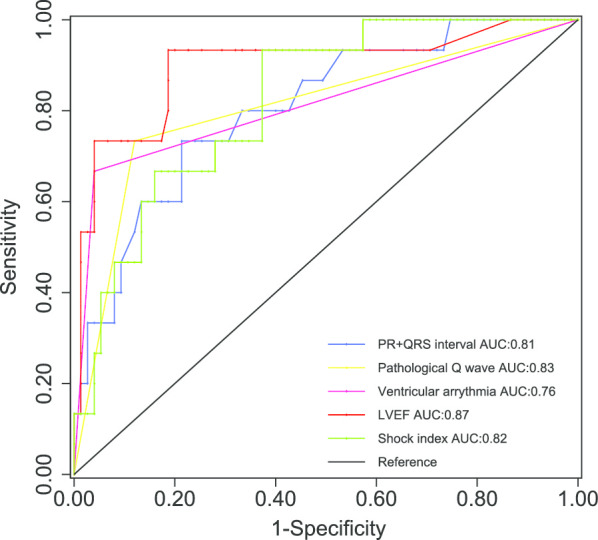
Comparison of the diagnostic capability for the different indices. There was no significant difference among the area under the five ROC curves

We performed the multivariate logistic regression for the eligible ECG parameters elected from the univariate analysis firstly in order to identify the independent relevant factors. For simplification, PR interval and QRS complex duration were added together to become a single variable: PR + QRS interval. This integrated variable together with pathological Q wave, ST segment elevation, ST segment depression, T wave inverse, ventricular arrhythmia and second to third degree AVB were set in the multivariate analysis. First degree AVB was excluded from multivariate analysis for sake of a certain overlap with PR interval. Consequently, PR + QRS interval, pathological Q wave and ventricular arrhythmia were confirmed as independent relevant factors (Table 4). Hosmer–Lemeshow χ^2^ test suggested a excellent model fit (χ^2^ = 1.92, *P* = 0.59). The diagnostic capability of the three factors together with LVEF and SI were evaluated using ROC (Fig. 2). LVEF had the largest AUC whereas ventricular arrhythmia had the smallest. Nevertheless, there was no significant difference in the AUC of the five indices.

**Table 4:** Multivariate analysis of ECG parameters for CS

Variables	Odd ratio	95% CI	*P* value
PR + QRS interval (per 10 ms increase)	1.29	[1.07 1.55]	0.008
Pathological Q wave	30.3	[4.40 208.1]	0.003
Ventricular arrhythmia	26.8	[2.99 238.6]	0.001

### Derivation of new predictor and model fit

**Fig. 3:**
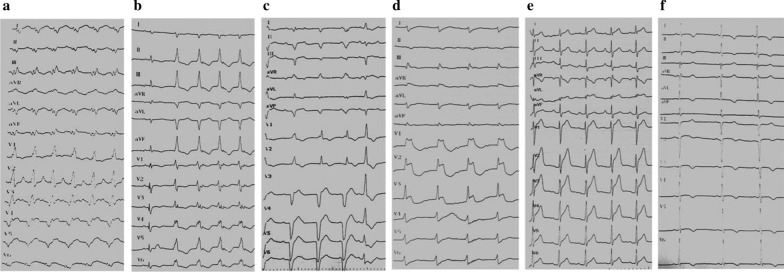
**a** FM patient. PR interval 150 ms, QRS complex 140 ms, with pathological Q wave, without ventricluar arrhythmia. ECG score = 580 points. **b** FM patient. PR interval 160 ms, QRS complex 130 ms, with ventricular tachycardia, without pathological Q wave. ECG score = 580 points. **c** FM patient. PR interval 170 ms, QRS complex 110 ms, with accelerated idoventricular rhythm, without pathological Q wave, ECG score = 560 points. **d** FM patient. PR interval 280 ms, QRS complex 240 ms, without ventricular arrhythmia and pathological Q wave. ECG score = 520 points. **e** NFM patient. PR interval 180 ms, QRS complex 100 ms, without ventricular arrhythmia and pathological Q wave. ECG score = 280 points. **f** NFM patient. PR interval 140 ms, QRS complex 90 ms, without ventricular arrhythmia and pathological Q wave. ECG score = 230 points

In order to promote the diagnostic capability of the three relevant factors elected from ECG, we conceived a new variable generated by a certain combination of the three variables. First, we denoted scores to each factor. PR interval and QRS duration were assigned the equivalent score to their actual values. For example, PR interval is given 150 points if it is 150 ms. QRS complex duration is given 140 points if it is 140 ms. PR + QRS interval is the sum of each score. Therefore, the score of PR + QRS interval in the above example is 290 points. Ventricular arrhythmia is denoted 1 point if it occurs while 0 point if it does not occur. Pathological Q wave is denoted the score in the same way. Second, we define the derived new variable. We call it “ECG score” for the present time and ECG score = (PR interval + QRS complex duration) × (ventricular arrhythmia + pathological Q wave + 1). For another example, if PR interval = 200 ms, QRS complex duration = 120 ms, ventricular tachycardia occurs without pathological Q waves existence, ECG score = (200 + 120) × (1 + 0 + 1) = 640 points. We provide some ECG of acute myocarditis in Fig. 3 to illustrate this method.

**Fig. 4:**
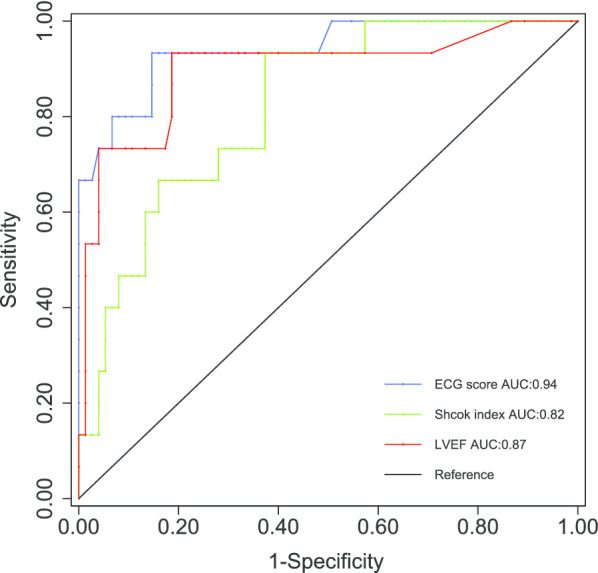
Assessment of the diagnostic capability of ECG score. The area under the ROC curve of ECG score was similar to that of LVEF, but significantly larger than that of shock index (*P* < 0.05)

Now the ECG score should be drawn in the multivariate regression model for assessing the odd ratio. The results of the model fit were listed in the Table 5. In the Model 1, ECG score and LVEF were identified as the significant relevant factors of CS (χ^2^ = 0.48, *P* = 0.92 for Hosmer–Lemeshow test). Model 2 was quite similar to the Model 1 but without LVEF. Consequently, ECG score and SI were the significant relevant factors in the Model 2 (χ^2^ = 1.68, *P* = 0.64 for Hosmer–Lemeshow test). The ROC of ECG score, LVEF and SI were depicted for comparing the diagnostic capability (Fig. 4). The AUC of ECG score was significantly larger than the AUC of SI (*P* < 0.05), but similar to that of LVEF. It indicated that ECG score seemed to be a more superior diagnostic index for CS.

**Table 5:** Multivariate regression model fit for the derived variable

Model	Variables	Odd ratio	95% CI	*P* value
Model 1 Age + Sex + SI + LVEF + IVST + PAP + ECG score	ECG Score (per 10 increase)	1.13	[1.07 1.09]	0.002
	LVEF (per 5% increase)	0.53	[0.33 0.85]	0.009
Model 2 Age + Sex + SI + IVST + PAP + ECG score	ECG Score (per 10 increase)	1.11	[1.06 1.18]	< 0.0001
	SI (per 0.1 increase)	1.42	[1.05 2.04]	0.049

*SI* shock index, *IVST* intraventricular septal thickness, *LVEF* left ventricular ejection fraction, *PAP* pulmonary artery pressure

### Cutpoint of the ECG score

The sensitivity, specificity, PPV, NPV and accuracy of the ECG score were listed in the Table 6. The identification of an appropriate cutoff value should consider the life-threatening situation of the CS, which needed a relative high NPV to reduce the false negative rate. ECG score below 500 points had quite a high NPV but PPV was a little more lower, which might increase the misdiagnosis. Thus, we set the suitable cutpoint of ECG score at 560 points with nearly 95% NPV and 74% PPV.

**Table 6:** Diagnostic capability of ECG score for CS at different cutpoints

Cutpoint	Sensitivity (%)	Specificity (%)	Accuracy (%)	PPV (%)	NPV (%)
400	94.4	82.9	85.1	58.6	98.4
440	94.4	85.5	87.2	59.3	95.6
480	83.3	86.8	86.2	65.2	95.8
520	83.3	89.5	88.3	65.2	95.8
560	77.8	93.4	90.4	73.7	94.7
600	66.7	96.1	90.4	84.6	91.4
640	61.1	97.4	90.4	91.7	91.5
680	55.6	100.0	91.5	100.0	90.1

*CS* cardiogenic shock, *PPV* positive predictive value, *NPV* negative predictive value

## Discussion

Acute myocarditis involves the degenerative or necrotic changes in the myocytes due to various etiologies. FM accounts for about 30% in the hospitalized patients of acute myocarditis [5]. Rapid progress of haemodymamical disorder and CS are the main characteristics of FM, which may lead to death in several hours or days if the patients are not treated efficiently. So far, the measurements for quick recognition of CS are limited. However, we have attempted to develop a new measurement using ECG to diagnose CS in the present of acute myocarditis.

In the acute phase, the depolarization, repolarization and electric conduction of the myocardial tissue will change to different extent. Sometimes the changes are too slight to be reflected on ECG. Many changes of ECG are nonspecific and not decisive for diagnosis. However, some morphological or electric alterations are more likely to occur in FM patients. ACS-like ECG usually includes pathological Q wave, ST segment elevation/depression and T wave inverse. The incidence of pathological Q wave varies in different studies [12, 13, 19]. In the current study, the incidence of pathological Q wave is 75% in FM group, which is higher than the previous studies. It is usually associated with the myocardial transmural lesion and correspond with the distribution of late gadolinium enhancement (LGE) on CMR [20, 21]. Furthermore, the presence of pathological Q wave is identified as a predictor of poor prognosis in FM patients [22]. ST-T alteration is present both in FM and NFM patients. We have reported a much higher incidence of ST segment elevation/depression and T wave inverse in FM group. Different from Q wave, ST segment elevation is less helpful in evaluating the location of myocardial tissue but can be used as a quick assessment of the extent of myocardial injury [23]. As to ST segment depression, it has been suggested an early sign of FM [15]. However, ST segment elevation/depression and T wave inverse were not identified relevant to the CS in the current study.

PR interval prolongation and wide QRS complex are suggestive of impairment of conduction system and the prevalence is usually in proportion to the severity of the conditions. We found that the PR interval and QRS complex duration in FM patients were both significantly prolonged than that in NFM patients. The incidence of wide QRS complex in our study was as high as 70%, which was very close to the previous findings [14]. And we also found these two index was useful in quick diagnosis of CS. Ventricular arrhythmia and AVB are both reported common in FM [14, 24], which has been supported by our findings. Nonetheless, tachyarrhythmia is more common than bradyarrhythmia [17]. Ventricular arrhythmia is considered related to myocardial edema, scar and severe impairment of cardiac function. LEG on CMR can increase the likelihood of ventricular arrhythmia [25, 26]. The current study found the ventricular arrhythmia were more likely to occur in FM patients but also occurred in a small part of NFM patients, which is consistent with the former study [24]. Ventricular arrhythmia, wide QRS complex and AVB are sometimes present in a single patient alternatively, even before the emergence of haemodynamical disorder. Seen in this light, certain morphological changes on ECG may be helpful to early and rapid recognition of CS.

The ECG score was generated completely original. PR interval and QRS complex duration are the main body of the score. Occurrence of ventricular arrhythmia and pathological Q wave will provide weight for the main body. It integrates different relevant factors of the ECG into a single index and can be used conveniently. ECG score is independent of LVEF and SI in diagnosing CS. It also presented a superior diagnostic capability in comparison with LVEF and SI. From Table 6, we found ECG score had a high NPV and therefore suitable for exclusion diagnosis. When ECG score was beyond 560 points, the PPV was increased significantly with a slight decrease of NPV. Of note, ECG score can be calculated only when sinus rhythm is seen on ECG for measurment of PR interval. If the ECG presents ventricular arrhythmia (such as ventricular tachycardia or AIVR) without sinus rhythm, it provides more powerful evidence for the diagnosis of fulminant myocarditis and ECG score is not necessary under this circumstance. Besides, subgroup analysis, such age subgroup, sex subgroup, was not performed due to relative small sample size. Thus, it was unclear whether the ECG score as a new diagnostic index was age specific or sex specific. We did even not know whether this score was applicable to the children because the study cohort did not contain children patients. However, these issues are quite important and meaningful. Particularly, it is worthy of further investigation whether the cutpoint varies in different age range or different sex.

In summary, the ECG score, derived from the parameters of ECG, have possessed an outstanding diagnostic capability of CS caused by acute myocarditis. It may be a reliable substitution for LVEF or SI and can be used for a early recognition of the haemodynamical derangement.

## Limitations

The current study is a single center and retrospective analysis. There are several limitations. First, the sample size is a little bit small. When we performed diagnostic statistics, there are breakpoints between the two adjacent cutoff values. We cannot learn the diagnostic indices at these breakpoints. Second, subgroup analysis could not be carried out due to the small sample size. Whether the ECG score also works in different age group or sex group is unknown. Whether the cutpoint varies in different subgoups is also unclear. Third, the serum lactate level is also an useful biomarker for early assessment of peripheral perfusion. Nonetheless, in our study, most of the haemodynamical stable patients did not have the lactate test. The comparison of ECG score with lactate could not be carried out. Fourth, endomyocardial biopsy was not available in our center. Despite all the patients were caused by infection, we could not evaluate the relationship between pathological type and severity of the conditions. The last but not least, the diagnostic capability of ECG score is acquired from the single center, retrospective study. We need multicenter, prospective trials with larger sample size to verify the conclusions.

## Data Availability

The information and data of the study population were acquired from Hospital Information System and were recorded manually in EXCEL to form the database. The datasets analyzed during the current study are not publicly available due to the protection of the individual privacy but are available from the corresponding author on reasonable request.

## Abbreviations

ACEI: Angiotensin-converting enzyme inhibitor
ACS: Acute coronary syndrome
AIVR: Accelerated idioventricular rhythm
ARB: Angiotensin receptor blockade
AUC: Area under curve
AVB: Atrioventricular block
BNP: Brain natriuretic peptide
CCU: Cardiac care unit
CHD: Coronary heart disease
CMR: Cardiac magnetic resonance
CS: Cardiogenic shock
ECG: Electrocardiography
ECMO: Extracorporeal membrane oxygenation
ED: Emergency department
FM: Fulminant myocarditis
FMC: First medical contact
IABP: Intra-aortic balloon pump
IQR: Interquartile range
IVST: Intraventricular septal thickness
LGE: Late gadolinium enhancement
LVEDD: Left ventricular end-diastolic diameter
LVEF: Left ventricular ejection fraction
LVESD: Left ventricular end-systolic diameter
LVPWT: Left ventricular posterior wall thickness
NFM: Non fulminant myocarditis
NPV: Negative predictive value
PPV: Positive predictive value
ROC: Receiver-operating characteristics
SI: Shock index
UCG: Ultracardiography
VT: Ventricular tachycardia
